# Optimal use of SGLT2 inhibitors in diabetic kidney transplant recipients

**DOI:** 10.3389/fneph.2022.1014241

**Published:** 2022-11-10

**Authors:** Phuong-Thu T. Pham, Phuong-Chi T. Pham

**Affiliations:** ^1^ Nephrology Division, Kidney Transplant Program David Geffen School of Medicine at University of California at Los Angeles (UCLA), Los Angeles, CA, United States; ^2^ Division of Nephrology and Hypertension Olive-View University of California at Los Angeles (UCLA) Medical Center, Sylmar, CA, United States; ^3^ David Geffen School of Medicine at University of California at Los Angeles (UCLA), Los Angeles, CA, United States

**Keywords:** sodium-glucose cotransporter 2 inhibitor, SGLT2 inhibitor, post-transplantation diabetes mellitus, PTDM, kidney transplantation, diabetic kidney disease, diabetic kidney transplant recipients

## Abstract

Sodium-glucose cotransporter 2 inhibitor (SGLT2i), a glucosuric agent initially approved for use as an antidiabetic agent, was unexpectedly found to confer cardio-and reno-protective effects in individuals with or without type 2 diabetes mellitus. Despite mounting evidence suggesting that SGLT2i provides cardio- and reno-protective benefits in both diabetic and non-diabetic and in chronic kidney disease (CKD) patients in the general population, reservations for its use in the transplant setting persist due to concerns for increased risk of genital mycotic and urinary tract infections. A comprehensive review of the literature on the efficacy and safety of SGLT2i use in diabetic kidney transplant recipients is herein presented followed by authors’ opinion on its optimal use in this patient population.

## Introduction

The sodium-glucose cotransporter 2 inhibitor antidiabetic drug class improves glucose control in diabetic patients by inducing glucosuria *via* inhibition of glucose reabsorption at proximal convoluted tubules. In patients without hyperglycemia, the glomerular filtered load of glucose is relatively low, thereby limiting the extent of glucosuria and inherently minimizes hypoglycemic risk (1). The cardiorenal protective effects of SGLT2i in subjects with or without type 2 diabetes mellitus have been demonstrated in several randomized controlled trials and systematic review and meta-analysis (2–6). The EMPEROR-Reduced randomized placebo-controlled trial designed to study the effect of empagliflozin on cardiovascular and kidney outcomes across the spectrum of kidney function demonstrated a significant reduction in cardiovascular death, heart failure hospitalization, and total heart failure hospitalization among empagliflozin-treated patients compared with their placebo-treated counterparts at a median follow-up of 16 months. A reduction in composite kidney outcome (defined as sustained profound decline in estimated glomerular filtration rate [eGFR], chronic dialysis, or transplant) was also observed among patients randomized to receive empagliflozin irrespective of baseline renal function (Hazard ratio [HR] for patients with vs. without chronic kidney disease: 0.53 vs. 0.46, respectively, p=0.78) (7). Whether the cardiorenal benefits of SGLT2i seen in the general population can be extrapolated to the transplant population remains to be elucidated. The following section provides a summary of clinical studies evaluating the efficacy and safety of SGLTi use in kidney transplant recipients with pre-existing diabetes or post-transplantation diabetes mellitus (PTDM).

## Methods

A literature search using PubMed Advanced Search Builder was conducted on October 1, 2022 using medical subject headings (MeSH) ([renal OR kidney] (1,246,987 articles) AND [transplant] (968,905) AND [sodium glucose transport protein 2, SGLT2, sodium glucose cotransporter 2, SGLT2, canaglifloxin, empagliflozin, ertugliflozin, OR dapagliflozin] (11,359)) yielded 223 articles. Full-text articles selected for inclusion in our review included randomized controlled trials, retrospective studies, cohort studies, and prospective and retrospective case series. A total of 10 studies evaluating the efficacy and safety of SGLT2i in diabetic kidney transplant recipients with pre-existing type 2 diabetes or PTDM were identified. Of the 10 studies, seven were included in a systematic review and meta-analysis (7/7 studies were graded as good quality using the quality assessment tool for case series studies proposed by the National Heart, Lung and Blood Institute). One additional study included in the systematic review and meta-analysis was in abstract format and was graded as fair quality.

## Studies evaluating the efficacy and safety of SGLT2i use in diabetic kidney transplant recipients

At the time of this writing, there has been only one prospective, randomized placebo-controlled trial evaluating the efficacy and safety of SGLT2i in kidney transplant recipients with post-transplantation diabetes mellitus (PTDM). Study inclusion criteria included more than one year after transplant, stable renal function (eGFR > 30 mL/min/1.73m^2^), and stable immunosuppressive therapy. Forty-four eligible patients with PTDM were randomized to receive either empagliflozin (n=22) or placebo (n=22) for 24 weeks. A statistically significant reduction in A1C and body weight was observed among empagliflozin-treated patients compared with their placebo-treated counterparts (-0.2% vs. 0.1%, p=0.025 and -2.5 kg vs. +1.0 kg, respectively, p=0.014). There were no significant differences in adverse events, immunosuppressive drug levels, or eGFR between the two treatment groups (8). The incidence of urinary tract infections (UTIs) was comparable between the two treatment arms. One patient in the empagliflozin group had genital mycotic infection. While randomized controlled trial provides the highest quality of evidence, the small sample size and short duration of follow-up limit the ability to evaluate the outcome of eGFR change.

Systematic review and meta-analysis of eight studies involving 132 diabetic kidney transplant recipients demonstrated that SGLT2i use resulted in a significantly lower A1C (-0.56%, p=0.007) and body weight (-2.16 kg, p < 0.001) at end of study compared with baseline levels (9–16). No significant changes in eGFR, serum creatinine, urine protein creatinine ratio, or blood pressure were observed. In total, fourteen of 132 study patients had urinary tract infections and one of 72 participants from 5 studies had genital mycosis. Other reported adverse events included one case of acute kidney injury (AKI), one case of small ulcer in the lower extremity, and one case of cellulitis. There were no reported cases of euglycemic ketoacidosis or acute rejection during the follow-up period. Of eight studies, one was randomized controlled trial, hence only patients in the treatment arm were included in the meta-analysis (8). Seven were categorized as case series. Baseline eGFR among all study patients was 64.5 ± 19.9 ml/min/1.72 m^2^. Mean time from transplantation varied from 3 to 20 years. Follow-up duration ranged from 6 to 24 months. Based on the study results, it was concluded that SGLT2i are effective in lowering A1C, reducing body weight, and preserving kidney function in diabetic kidney transplant recipients without serious adverse events including euglycemic ketoacidosis and acute rejection (16).

In one single-center retrospective study designed to evaluate the metabolic, electrolyte and safety outcomes of early SGLT2i utilization in diabetic kidney transplant recipients, a significant reduction in body weight (-2.95 kg, p < 0.0001) and improvement in magnesium concentration (0.13, p=0.0004) were observed at 6-month follow-up. Overall insulin requirements decreased by -3.7 units (SD 22.8, p=0.17). There was no statistically significant change in eGFR at three and six months after SGLT2i initiation (change in eGFR of -1 mL/min/1.73m^2^, p=0.8831 and 1 mL/min/1.73m^2^, p=0.1478, respectively). Of the 50 patients enrolled in the study, half were started on therapy within the first year after transplant with a median time to drug initiation of 319 days. The incidence of UTIs was not significantly different from that reported historically in this high-risk patient population (14% vs. ~ 20%, respectively). There was one case of genital yeast infection. No cases of diabetic ketoacidosis (DKA), amputations, or AKI were seen. Study inclusion criteria included absence of AKI within 30 days prior to the initiation of SGLT2i, freedom from any UTIs 6 months prior to SGLT2i initiation, and eGFR ≥ 30 mL/min/1.73m^2^ at the time of SGLT2i initiation (17).

A retrospective multicenter study aimed to assess the safety, tolerability, and effectiveness of SGLT2 in kidney transplant recipients with pre-existing type 2 DM or PTDM demonstrated that adding SGLT2i to a regimen containing three antihyperglycemic agents resulted in a modest reduction in A1C at 3- and 12- month follow-up (0.6% [p=0.013] and 0.4% [p=0.016], respectively). Non-statistically significant weight loss occurred in 15 patients with a recorded weight at both baseline and 3 months post SGLT2i initiation (weight loss of 1.6 kg, p=0.11). Median time from kidney transplant to SGLT2i initiation was 28 months (range 16-60 months). Of 39 patients studied, 27 remained on therapy for at least 1 year. In patients with follow-up data, no statistically significant changes in kidney function (n=35) or tacrolimus exposure was observed (n=24). Ten patients (25%) experienced an adverse event while on SGLT2i therapy, with UTI being the most common (n=6, three required hospitalization). Five of six patients with UTI had a history of UTI prior to SGLT2 initiation. Four continued therapy without UTI recurrence. Urosepsis and pyelonephritis leading to SGLT2i discontinuation occurred in patients with prior history of recurrent UTIs. One patient developed diabetic ketoacidosis (due to uncontrolled diabetes) and concurrent UTI. Two patients developed complications from diabetic foot ulcers, both of whom had known peripheral vascular disease and prior history of diabetic foot ulcer. Two patients developed AKI more than 90 days after SGLT2i initiation determined by independent investigators to be unrelated to SGLT2i therapy. No episode of Fournier gangrene, genital mycotic infection, or bone fractures were identified during the study period (18).

A retrospective multicenter cohort study conducted to evaluate the efficacy and safety of SGLT2i in kidney transplant recipients with either pre-existing or post-transplantation diabetes demonstrated that SGLT2i use confers a renoprotective effect. Of the 2083 diabetics enrolled in the study, 226 were prescribed SGLT2i for more than 90 days and were included in the study. Baseline eGFR were similar between SGLT2i users and non-users (69.8 ± 17.8 vs. 68.9 ± 20.4 mL/min/1.73m^2^, respectively, p=0.459). During a mean follow-up of 62.9 ± 42.2 months, a significantly lower risk of the primary composite outcome of all-cause mortality, death-censored graft failure, and serum creatinine doubling were observed among SGLT2i users compared with non-users in multivariate and propensity score-matched models (adjusted HR 0.43; p=0.006, and adjusted HR 0.45; p=0.013, respectively). All three individual components of the outcome were significantly lower in SGLT2i users compared with non-users. A transient dip of more than 10% decline in eGFR occurred in 15.6% of SGLT2i-treated group during the first month but gradually recovered thereafter. Risk factors for eGFR dip were shorter time from transplantation to SGLT2i usage and higher mean tacrolimus trough levels. *Post hoc* analysis showed no difference in eGFR between the dipper and non-dipper groups at all time points. A significant decrease in urine protein-to-creatinine ratio was observed in both dipper (p=0.003) and non-dipper groups (p < 0.001). The incidence of any bacterial or fungal urinary tract infections were similar between SGLT2i users and non-users and no case of euglycemic ketoacidosis was identified. The study findings suggest that SGLT2i confers a renoprotective effect in diabetic kidney transplant recipients and its use is safe in such population (19).

However, the study is not without potential confounding factors. Notably, patients who were prescribed SGLT2i for less than 90 days were excluded from the study analysis (n=29), raising concerns that SGLT2i adverse events or deterioration in allograft function may have led to the discontinuation of therapy in these patients (20). Nonetheless, data from clinical trials and propensity-matched analyses of data from clinical practice unequivocally demonstrated that SGLT2i are “kidney safe” and do not predispose to acute kidney injury (AKI) (21). In contrast, its use reduces AKI risk and protects against diabetic kidney disease progression in the non-transplant population (6, 21).

Angiotensin-converting enzyme inhibitor (ACEI) or angiotensin-receptor blocker (ARB) use was more common in SGLT2i users vs. non-users (48.7% vs. 33.8%, respectively, p < 0.001). Nonetheless, although the cardiorenal protective effects of ACEI and ARB have been well-described in the general population, their favorable impact on patient and graft survival in the transplant setting has not been consistently demonstrated (22).

Studies evaluating the efficacy and safety of SGLT2i use in diabetic kidney transplant recipients are summarized in **Table 1**.

**Table 1:** Studies evaluating the efficacy and safety of SGLT2i use in diabetic kidney transplant recipients.

Study type	Aim of study	Efficacy/Beneficial effects	Safety
Randomized controlled trial (Halden et al) N= 44 randomized to receive either empagliflozin or placebo for 24 wk	To evaluate the efficacy and safety of SGLT2i in kidney transplant recipients with PTDM	AlC in empagliflozin-treated vs. placebo-treated­ treated patients (-0.2% vs. 0.1%, p=0.025) Body weight in empagliflozin-treated vs. placebo-treated patients (-2.5 kg vs. +1.0 kg, respectively, p=0.014)	Adverse events, immunosuppressive drug levels, eGFR,incidence of UTIs were comparable between the two treatment groups. One patient in the empagliflozin group had genital mycotic infection
Systematic review and meta-analysis (Chewcharat et al) N=132	To evaluate the efficacy and safety of SGLT2i for the treatment of DM among kidney transplant recipients	SGLT2i use resulted in a significantly lower AlC (-0.56%, p=0.007) and body weight (-2.16 kg, p<0.001) at end of study compared with baseline levels	No significant changes in eGFR, serum creatinine, urine protein creatinine ratio, or blood pressure were observed
Single-center retrospective study (Song et al) N=50	To evaluate the metabolic, electrolyte and safety outcomes of early SGLT2i utilization in diabetic kidney transplant recipients	A significant reduction in body weight (-2.95 kg, p<0.0001) and improvement in magnesium concentration (0.13, p=0.0004) were observed at 6-m follow-up Overall insulin requirements decreased by -3.7 units (SD 22.8, p=0.17)	Changes in eGFR at 3- and 6-m after SGLT2i initiation (eGFR of -1 mL/min/1.73m^2^, p=0.8831 and 1mL/min/1.73m^2^, p=0.1478, respectively). The incidence of UTIs was not significantly different from that reported historically (14% vs. 20%, respectively) There was one case of genital yeast infection. No cases of DKA, amputations, or AKI were observed
Retrospective multicenter study (Lemke et al) N=39	To assess the safety, tolerability, and effectiveness of SGLT2i after kidney transplantation	Modest reduction in AlC at 3- and 12-m (0.6% [p=0.013] and 0.4% [p=0.016], respectively). Non-statistically significant weight loss at 3 m post SGLT2i initiation (-1.6 kg, p= 0.11)	No significant changes in kidney function or tacrolimus exposure were observed Adverse event occurred in 10 patients with UTI being the most common (n=6). Five of six had a h/o UTI prior to SGLT2i initiation. Urosepsis and pyelonephritis occurred in patients with prior h/o recurrent UTIs. One patient developed DKA (due to uncontrolled diabetes) and concurrent UTI. Two patients developed complications from diabetic foot ulcers (both with h/o of PVD and prior h/o diabetic foot ulcer).
Retrospective multicenter cohort study (Lim et al) **N=** 226 were prescribed SGLT2i for> 90 d	To evaluate the efficacy and safety of SGLT2i in kidney transplant recipients with either pre­ existing or PTDM	Risk of primary composite outcome of all-cause mortality, death-censored graft failure, and serum creatinine doubling was significantly lower among SGLT2i users compared with non­ users in multivariate and propensity score­ matched models at a mean follow-up of 62.9 ± 42.2 m (adjusted HR 0.43; p=0.006, and adjusted HR 0.45; p=0.013, respectively)	A transient dip of > 10% decline in eGFR occurred in 15.6% of SGLT2i-treated group during the first month but gradually recovered thereafter. The incidence of any bacterial or fungal UTIs were similar between SGLT2i users and non-users. No case of euglycemic ketoacidosis was identified

SGLT2i, sodium-glucose cotransporter 2 inhibitor; eGFR, estimated glomerular filtration rate; UTI, urinary tract infection; AKI, acute kidney injury; h/o, history of; DKA, diabetic ketoacidosis.

## Discussion

SGLT2i use in diabetic kidney transplant recipients results in modest decrease in A1c without increased risk for genitourinary infectious complications or euglycemic DKA. However, it should also be noted that patients with history of recurrent UTIs, urosepsis or genital mycotic infections were generally excluded from these studies. Furthermore, in most studies, patients selected for SGLT2i therapy were more than 1-year post-transplantation because of concerns for fluctuating kidney allograft function and increased infection risk in the early post-transplantation period. Nonetheless, the potential beneficial effects of SGLT2i including weight loss, renoprotection, metabolic and electrolyte benefit, and lack of significant drug-drug interaction with calcineurin inhibitor render SGLT2i an attractive treatment option for kidney transplant recipients. Of interest, an experimental animal model of tacrolimus-induced diabetes demonstrated that empagliflozin improves hyperglycemia and suppressed the tacrolimus-induced twofold increase in the expression of SGLT2 (23). Furthermore, empagliflozin was found to have a direct protective effect on tacrolimus-induced renal injury. The study findings suggest that SGLT2i is suitable for use in kidney transplant recipients with tacrolimus-induced PTDM.

Similar to the general population, the use of SGLT2i in the transplant setting can cause an acute transient dip in eGFR, thought to be due to SGLT2i induced afferent arteriolar vasoconstriction. It is suggested that the natriuretic effect of SGLT2i leads to increased tubuloglomerular feedback and afferent arteriolar vasoconstriction even in denervated kidney allograft. Whether the resultant reduction in intraglomerular hypertension and hyperfiltration play a role in reducing proteinuria and preserving eGFR in diabetic kidney transplant recipients warrants further investigation.

To date, there has been no case of Fournier gangrene associated with SGLT2i use in diabetic kidney transplant recipients. One case of new diabetic foot ulcer/osteomyelitis occurring years after starting empagliflozin leading to distal limb amputation has been reported. Notably, the patient had a history of peripheral vascular disease and prior history diabetic foot ulcers (18). It has been hypothesized that the diuretic effect of SGLT2i could result in hypovolemia and hypoperfusion of distal lower-extremities, potentially leading to limb ischemia and necrosis and ultimately limb amputation (24). Clinicians should remain vigilant to such complications particularly in high risk diabetics with pre-existing foot ulcers or peripheral arterial disease.

In the authors’ opinion, SGLT2i use in diabetic kidney transplant recipients appears safe and effective in selected candidates. Based on currently available literature, optimal use of SGLT2i in kidney transplant recipients with pre-existing diabetes or PTDM is suggested in **Table 2** (12, 19, 25–29). The routine recommendation for SGLT2i use in diabetic and non-diabetic kidney transplant recipients for their potential cardio- and reno-protective effects awaits further studies. Large randomized controlled trials with long-term follow-up are needed.

**Table 2:** Optimal use of SGLT2i in kidney transplant recipients with pre-existing DM or PTDM.

General comments about SGLT2i	•2022 ADA-KDIGO approach for improving outcomes in patients with type 2 DM and CKD (non-transplant population): •First line drug therapy: initiate SGLT2i if eGFR≥20 ml/min; continue until dialysis or transplant *and/or* initiate metformin if eGFR≥30 ml/min •There has been no consensus guideline on the use of SGLT2i in diabetic kidney transplant recipients. Authors' opinion based on currently available safety and efficacy data: •Consider first line therapy if A1C is not too far from goal or as add-on therapy to keep A1C at goal (generally < 7%) •Potential beneficial effect in patients with micro- or macroalbuminuria •Major effect: •Modest reduction in A1C •Not effective for glycemic control if eGFR < 30-45 ml/min (may use solely for potential cardiorenoprotective effects)^1^ •Miscellaneous effects •Blood pressure lowering •Weight loss •Increase serum magnesium levels (potential benefit in calcineurin inhibitor-induced hypomagnesemia) •Reduction in urine protein creatinine ratio and albuminuria^2^ •Uricosuria •Increase hemoglobin and hematocrit levels^3^ •*In vitro* studies suggest that canagliflozin has anti-inflammatory and antifibrotic effects in diabetic kidney disease^4^ •Canagliflozin (FDA warning: Increased risk for bone fractures and decreased bone mineral density)
Diabetic kidney transplant recipient: SGLT2i candidate (Must meet all criteria)	•At least 6-12 m after transplant with stable graft function for at least 4 w •No UTI or genital mycotic infection within 6 m •No acute rejection within 6 m •On stable dose of immunosuppression •No requirement for intensification of immunosuppression within 6 m
Use with caution	•Concomitant diuretic use: May need to adjust diuretic dose to minimize risk of volume depletion •Caloric malnutrition •Elderly: Need to closely assess volume and nutritional status •Alcohol abuse (malnutrition and increased DKA risk)
Hold	•Pre-operative: Hold at least 3 to 4 days before any scheduled surgery that requires NPO to avoid the potential risk for DKA^5^ •Poor oral intake, nausea, vomiting, diarrhea, hypotension •Prolonged fasting (increased DKA risk)
Contraindications or avoid	•Type 1DM, history of DKA or euglycemic DKA •Patients at risk for or with a history of recurrent genital mycotic infections, UTIs, pyelonephritis or urosepsis •Neurogenic bladder, vesicoureteral reflux, urinary incontinence •Indwelling Foley catheter, clean intermittent catheterization, or presence of ureteral stent •Asymptomatic chronic bacteriuria or pyuria (safety data lacking) •History of recurrent UTIs or pyelonephritis in native kidneys (safety data lacking) •Chronic hypotension, recurrent volume depletion •Patients at risk for or with a history of lower limb amputation, risk factors for Fournier's gangrene, or peripheral vascular disease •Recurrent pancreatitis

^1^Renal dose adjustment required for specific SGLT2i types.

^2^References 12, 25-27.

^3^In type 2 DM, hyperglycemia may alter hypoxia-inducible factor pathways and impair erythropoiesis. It is speculated that by inhibiting glucose reabsorption, SGLT2 inhibitors may attenuate glucotoxicity in renal tubulointerstitium, thereby allowing renal erythropoietin-producing cells to resume their function and increase EPO secretion. PMID: 32241009 (reference 28).

^4^PMID: 31001673 (reference 29).

^5^Empa-, dapa-, and canagliflozin: Hold for at least 3 days prior to scheduled surgery; Ertugliflozin: Hold for at least 4 days prior to scheduled surgery (see package insert)

SGLT2i, sodium-glucose cotransporter 2 inhibitor; ADA, American Diabetes Association; KDIGO, Kidney disease improving global outcomes; DM, diabetes mellitus; CKD, chronic kidney disease; eGFR, estimated glomerular filtration rate; FDA, Federal Drug and Food Administration; DKA, diabetic ketoacidosis; NPO, nothing by mouth; UTIs, urinary tract infections.

## Author contributions

P-PT and P-PC contributed equally to searching the literature, writing the manuscript, and designing the tables. All authors contributed to the article and approved the submitted version.

## Conflict of interest

The authors declare that the research was conducted in the absence of any commercial or financial relationships that could be construed as a potential conflict of interest.

## Publisher’s note

All claims expressed in this article are solely those of the authors and do not necessarily represent those of their affiliated organizations, or those of the publisher, the editors and the reviewers. Any product that may be evaluated in this article, or claim that may be made by its manufacturer, is not guaranteed or endorsed by the publisher.

## References

[1] Heerspink HJL Perkins BA Fitchett DH Husain M Cherney DZI. Sodium glucose cotransporter 2 inhibitors in the treatment of diabetes mellitus. Circulation (2016) 134:752–72. doi: 10.1161/CIRCULATIONAHA.116.021887 27470878

[2] Zelniker TA Wiviott SD Raz I Im K Goodrich EL Bonaca MP. SGLT2 inhibitors for primary and secondary prevention of cardiovascular and renal outcomes in type 2 diabetes: A systematic review and meta-analysis of cardiovascular outcome trials. Lancet (2019) 393(10166):31–9. doi: 10.1016/S0140-6736(18)32590-X 30424892

[3] Zelniker TA Braunwald E. Mechanisms of cardiorenal effects of sodium-glucose cotransporter 2 inhibitors: JACC state-of-the-Art review. J Am Coll Cardiol (2020) 75(4):422–34. doi: 10.1016/j.jacc.2019.11.031 32000955

[4] Anker SD Butker J Filippatos G Ferreira JP Bocchi E Böhm M. Empagliflozin in heart failure with a preserved ejection fraction. N Engl J Med (2021) 385(16):1451–61. doi: 10.1056/NEJMoa2107038 34449189

[5] Neuen BL Ohkuma T Neal B Matthews DR de Zeeuw D Mahaffey KW. Relative and absolute risk reductions in cardiovascular and kidney outcomes with canagliflozin across KDIGO risk categories: Findings from the CANVAS program. Am J Kidney Dis (2021) 77(1):23–34.e1. doi: 10.1053/j.ajkd.2020.06.018 32971190

[6] Neuen BL Young T Heerspink HJL Neal B Perkovic V Billot L. SGLT2 inhibitors for the prevention of kidney failure in patients with type 2 diabetes: A systematic review and meta-analysis. Lancet Diabetes Endocrinol (2019) 7(11):845–54. doi: 10.1016/S2213-8587(19)30256-6 31495651

[7] Zannad F Ferreira JP Pocock SJ Zeller C Anker SD Butler J. Cardiac and kidney benefits of empagliflozin in heart failure across the spectrum of kidney function: Insights from EMPEROR-reduced. Circulation (2021) 143(4):310–21. doi: 10.1161/CIRCULATIONAHA.120.051685 PMC7834910 33095032

[8] Halden TAS Kvitne KE Midtvedt K Rajakumar L Robertsen I Jan Brox J. Efficacy and safety of empagliflozin in renal transplant recipients with posttransplant diabetes mellitus. Diabetes Care (2019) 42(6):1067–74. doi: 10.2337/dc19-0093 30862658

[9] Rajasekeran H Kim SJ Cardella CJ Schiff J Cattral M Cherney DZI. Use of canagliflozin in kidney transplant recipients for the treatment of type 2 diabetes: A case series. Diabetes Care (2017) 40(7):e75–6. doi: 10.2337/dc17-0237 28416475

[10] Schwaiger E Burghart L Signorini L Ristl R Kopecky C Tura A. Empagliflozin in posttransplantation diabetes mellitus: A prospective, interventional pilot study on glucose metabolism, fluid volume, and patient safety. Am J Transplant (2019) 19(3):907–19. doi: 10.1111/ajt.15223 PMC6590167 30585690

[11] Shah M Virani Z Rajput P Shah B. Efficacy and safety of canagliflozin in kidney transplant patients. Indian J Nephrol (2019) 29(4):278–81. doi: 10.4103/ijn.IJN_2_18 PMC6668319 31423063

[12] Attallah N Yassine L. Use of empagliflozin in recipients of kidney transplant: A report of 8 cases. Transplant Proc (2019) 51(10):3275–80. doi: 10.1016/j.transproceed.2019.05.023 31732204

[13] Mahling M Schork A Nadalin S Fritsche A Heyne N Guthoff M. Sodium-glucose cotransporter 2 (SGLT2) inhibition in kidney transplant recipients with diabetes mellitus. Kidney Blood Press Res (2019) 44(5):984–92. doi: 10.1159/000501854 31437852

[14] Kong J Joon J Chul Y Eun W Hyuk K Hyun SS. Sodium/glucose cotransporter 2 inhibitor for the treatment of diabetes in kidney transplant patients. Nephrol Dial Transplant (2019) 34:SP770.

[15] Alkindi f Al-Omary HL Hussain Q Hakim MA Chaaban A Boobes Y. Outcomes of SGLT2 inhibitors use in diabetic renal transplant patients. Transplant Proc (2020) 52(1):175–8. doi: 10.1016/j.transproceed.2019.11.007 31924404

[16] Chewcharat A Prasitlumkum N Thongprayoon C Bathini T Medaura J Vallabhajosuyula S. Efficacy and safety of SGLT-2 inhibitors for treatment of diabetes mellitus among kidney transplant patients: A systematic review and meta-analysis. Med Sci (Basel) (2020) 8(4):47. doi: 10.3390/medsci8040047 33213078 PMC7712903

[17] Song CC Brown A Winstead R Yakubu I Demehin M Kumar D. Early initiation of sodium-glucose linked transporter inhibitors (SGLT-2i) and associated metabolic and electrolyte outcomes in diabetic kidney transplant recipients. Endocrinol Diabetes Metab (2020) 4(2):e00185. doi: 10.1002/edm2.185 33855198 PMC8029504

[18] Lemke A Brokmeier HM Leung SB Mara KC Mour GK Wadei HM. Sodium-glucose cotransporter 2 inhibitors for treatment of diabetes mellitus after kidney transplantation. Clin Transplant (2022) 36(8):e14718. doi: 10.1111/ctr.14718 35593882

[19] Lim JH Kwon S Jeon Y Kim YH Kwon H Kim YS. The efficacy and safety of SGLT2 inhibitor in diabetic kidney transplant recipients. Transplantation (2022) 106(9):e404–12. doi: 10.1097/TP.0000000000004228 35768908

[20] Francis RS. SGLT2i after kidney transplantation: Ready for prime time? Transplantation (2022) 106(9):1732–3. doi: 10.1097/TP.0000000000004227 35777737

[21] Sridhar VS Tuttle KR Cherney DZI. We can finally stop worrying about SGLT2 inhibitors and acute kidney injury. Am J Kidney Dis (2020) 76(4):454–6. doi: 10.1053/j.ajkd.2020.05.014 32712014

[22] Hiremath S Fergusson DA Fergusson N Bennett A Knoll GA. Renin-angiotensin system blockade and long-term clinical outcomes in kidney transplant recipients: A meta-analysis of randomized controlled trials. Am J Kidney Dis (2017) 69(1):78–86. doi: 10.1053/j.ajkd.2016.08.018. 27712852

[23] Jin J Jin L Luo K Lim SW Chung BH Yang CW. Effect of empagliflozin on tacrolimus-induced pancreas islet dysfunction and renal injury. Am J Transplant (2017) 17(10):2601–16. doi: 10.1111/ajt.14316 28422431

[24] Potier L Mohammedi K Velho G Roussel R. SGLT2 inhibitors and lower limb complications: The diuretic-induced hypovolemia hypothesis. Cardiovasc Diabetol (2021) 20:107. doi: 10.1186/s12933-021-01301-x 33985506 PMC8120812

[25] Mahmoud TS Yagan J Hasan A Gheith O Mostafa M Rida S. Outcomes of sodium-glucose cotransporter 2 inhibitors and glucagon-like peptide-1 receptor agonists in diabetic kidney transplant recipients. Am J Transplant (2022) 22(supl 3). Available at: https://atcmeetingabstracts.com/abstract/outcomes-of-sodium-glucose-cotransporter-2-inhibitors-and-glucagon-like-peptide-1-receptor-agonists-in-diabetic-kidney-transplant-recipients/ 10.1111/ctr.15144 37755118

[26] Song C Brown A Winstead R Sterling S Yakubu I Christensen J. Intermediate term outcomes of SGLT2 inhibitors amongst diabetic kidney transplant recipients. Am J Transplant (2022) 22(suppl 3). Available at: https://atcmeetingabstracts.com/abstract/intermediate-term-outcomes-of-sglt2-inhibitors-amongst-diabetic-kidney-transplant-recipients/

[27] Yagan J Mahmoud TS Hasan A Gheith O El-Serwy n Rida S. Sodium-glucose Co-transporter 2 inhibitors; short-term outcome in diabetic kidney transplant recipients. Am J Transplant (2022) 22(suppl 3). Available at: https://atcmeetingabstracts.com/abstract/outcomes-of-sodium-glucose-cotransporter-2-inhibitors-and-glucagon-like-peptide-1-receptor-agonists-in-diabetic-kidney-transplant-recipients 10.1111/ctr.15144 37755118

[28] Marathias KP Lambadiari VA Markakis KP Vlahakos VD Bacharaki D Raptis AE. Competing effects of renin angiotensin system blockade and sodium-glucose cotransporter-2 inhibitors on erythropoietin secretion in diabetes. Am J Nephrol (2020) 51(5):349–56. doi: 10.1159/000507272 32241009

[29] Heerspink HJL Perco P Mulder S Leierer J Hansen MK Heinzel A. Canagliflozin reduces inflammation and fibrosis biomarkers: A potential mechanism of action for beneficial effects of SGLT2 inhibitors in diabetic kidney disease. Diabetologia (2019) 62(7):1154–66. doi: 10.1007/s00125-019-4859-4 PMC6560022 31001673

